# Influence of Root Color and Tissue on Phytochemical Contents and Antioxidant Activities in Carrot Genotypes

**DOI:** 10.3390/foods12010120

**Published:** 2022-12-26

**Authors:** Shiva Ram Bhandari, Chang Sun Choi, Juhee Rhee, Yu Kyeong Shin, Jae Woo Song, Seong-Hoon Kim, Solly Kang, Jun Gu Lee

**Affiliations:** 1 Department of Horticulture, College of Agriculture & Life Sciences, Jeonbuk National University, Jeonju 54896, Republic of Korea; 2 Core Research Institute of Intelligent Robots, Jeonbuk National University, Jeonju 54896, Republic of Korea; 3 Breeding Research Institute, Koregon Co., Ltd., Gimje 54324, Republic of Korea; 4 National Agrobiodiversity Center, National Institute of Agricultural Sciences, Rural Development Administration, Jeonju 54874, Republic of Korea; 5 Institute of Agricultural Science & Technology, Jeonbuk National University, Jeonju 54896, Republic of Korea

**Keywords:** carrot, carotenoid, outer and inner tissue, phytosterols, root color, total anthocyanin, vitamins

## Abstract

This study monitored changes in major carotenoids (lutein, ⍺-carotene, and β-carotene), free sugars (fructose, glucose, and sucrose), ascorbic acid, vitamin E, phytosterols (campesterol, stigmasterol, and β-sitosterol), fatty acid composition, total phenol content (TPC), total flavonoid content (TFC), total anthocyanin content, and antioxidant activities (AA); ferric-reducing antioxidant power (FRAP) and 2,2′-azino-bis (3-ethylbenzothiazoline-6sulfonic acid) [ABTS] assays, in the inner and outer root tissues of nine carrot genotypes with orange, white, and purple roots. The results showed a differential accumulation of bioactive compounds and antioxidant activities depending on root tissue and color. Carotenoids, free sugars, and total phytosterol contents were higher in genotypes with orange roots than in other genotypes. Ascorbic acid, TPC, TFC, total anthocyanin, and AA were highest in purple-colored carrots while vitamin E content was higher in white/purple carrots. Root color was highly related to the accumulation of individual carotenoids, vitamin E isomers, and total anthocyanin content most prominently among the analyzed bioactive compounds and AA. Free sugar and carotenoid contents were relatively higher in outer tissues than in inner tissues. Furthermore, ascorbic acid, TPC, TFC, and AA were statistically higher or similar in outer tissues when compared to inner tissues in all genotypes. In contrast, trends in vitamin E and phytosterol content were inconsistent between the inner and outer tissues, depending on the genotype. Although fatty acid composition was affected by both root color and tissue, the results were not significant. Thus, the phytochemical profile and content were highly dependent on root color and tissue in carrot genotypes. This may be useful in the food processing and pharmaceutical industries for the extraction of targeted bioactive compounds.

## 1. Introduction

Carrots (*Daucus carota* L.; 2*n* = 18) are one of the most important root vegetables grown worldwide, ranking as one of the 10 most cultivated crops in the world with a total cultivation area of 1.13 million ha and a worldwide annual production (carrots and turnips) of ~41 million tons [1]. Several epidemiological studies have shown that increased consumption of carrots is associated with a reduced risk of cancer, chronic diseases, cardiovascular diseases, and age-related macular degeneration. The health benefits of carrots are mainly attributed to the presence of a wide range of bioactive compounds, including carotenoids, phenolics, flavonoids, vitamins, anthocyanins, minerals, and fatty acids [2,3,4,5]. Carotenoids are one of the most important phytochemicals in carrots, with α- and β-carotene being the most abundant, accounting for 80–90% of the total carotenoid content in most carrot genotypes [6], although their composition is highly dependent on root color and genotype [7,8]. In general, orange carrots have high levels of α- and β-carotene, whereas purple carrots have high anthocyanin and polyphenol content [7,8]. Carotenoids protect DNA and proteins and reduce the risk of non-alcoholic fatty liver disease (NAFLD), heart diseases, and lipid oxidation, prevent cancer, and maintain normal vision [6,9,10,11,12,13,14,15]. Carotenoids are associated with a reduced risk of age-related macular degeneration, cataracts, and coronary heart disease [3,16]. Vitamins (vitamins C and E) are important phytochemicals found in carrots and exhibit antioxidant, anti-proliferative, and anticancer activities [17]. Among the vitamin E isomers, α-tocopherol is the most abundant in carrots, while the levels of other vitamin E isomers are highly dependent on the genotype [18]. Carrot roots also possess considerable amounts of phytosterols, conferring them with high health potential [19]. Carbohydrates are the primary compounds involved in plant metabolism and can be used as energy sources for plant growth and development. Free sugars are responsible for increasing the palatability and determining the sweetness of fruits and vegetables, which in turn alters the flavor, acceptability, and perception of flavor sensations [20,21]. Among the three major carbohydrates (sucrose, glucose, and fructose) present in carrot roots, sucrose is the most abundant [22], but its proportion is influenced by both genotype and environmental factors [4,23,24]. Anthocyanins, which are generally found in red and purple carrots, exhibit antioxidant and anticancer activities [25].

The functional quality and composition of bioactive compounds in carrots are significantly influenced by genetic and environmental factors (developmental stage, climatic conditions, growing seasons, agricultural practices, and postharvest storage conditions) and root color [2,5,7,8,24,26,27,28,29], altering the accumulation of bioactive compounds and their health potential [5,7,8]. The composition of bioactive compounds and their availability in carrot roots may be altered in carrot tissues, as the outer tissue (epidermis and pericarp) and inner tissue (xylem and pith) have different functions in the translocation of compounds. However, research related to the accumulation and availability of major bioactive compounds in the inner and outer tissues of carrot root is limited. Therefore, this study aimed to compare the biochemical constituents (carotenoids, vitamins C and E, total phenol, total anthocyanin, total flavonoid, free sugars, phytosterols, and fatty acids) and antioxidant activities (ABTS and FRAP assays) in the inner and outer tissues of nine carrot genotypes with different root colors. The results of this study might be useful for plant breeders, food industries, and consumers. Insight into the major bioactive compounds in different tissues and plant genotypes might help to characterize their chemical composition and the information on the tissue-dependent accumulation of bioactive compounds could be used for the processing and extraction of targeted bioactive compounds from the tissues of given genotypes.

## 2. Materials and Methods

### 2.1. Chemicals and Reagents

Authentic standards for carotenoids (lutein, α-carotene, and β-carotene), free sugars (glucose, fructose, and sucrose), ascorbic acid, and phytosterols (campesterol, β-sitosterol, and stigmasterol) were purchased from Sigma-Aldrich (St. Louis, MO, USA). Fatty acid methyl esters were obtained from Supleco (Bellefonte, PA). Vitamin E standards were purchased from Merck (Darmstadt, Germany). High-performance liquid chromatography (HPLC) grade methanol, acetonitrile, ethanol, chloroform, n-hexane, iso-octane (2,2,4-trimethyl pentane), benzene, hydrochloric acid (HCl), and water were obtained from Avantor Performance Materials (Center Valley, PA, USA). Metaphosphoric acid, formic acid, acetic acid, Folin’s reagent, sodium carbonate (Na_2_CO_3_), gallic acid, aluminum chloride hexahydrate (AlCl_3_6H_2_O), sodium nitrite (NaNO_2_), sodium hydroxide (NaOH), catechin hydrate, 2,2-dimethoxy propane, n-heptane, sulfuric acid (H_2_SO_4_), anhydrous sodium sulfate (Na_2_SO_4_), acetic acid, ferric chloride hexahydrate (FeCl_3_6H_2_O), potassium hydroxide (KOH), sodium acetate trihydrate (C_2_H_3_NaO_2_3H_2_O), (±)-6-hydroxy-2,5,7,8-tetramethylchromane-2-carboxylic acid (Trolox), 2,4,6-Tris(2-pyridyl)-s-triazine (TPTZ), and 2,2′-azino-bis(3-ethylbenzothiazoline-6-sulfonic acid) diammonium salt were obtained from Sigma-Aldrich.

### 2.2. Plant Material and Cultivation of Carrot

Nine carrot genotypes with different root colors (orange, white, and purple) were used in this study. Seeds were provided by the National Agrobiodiversity Center, Jeonju, Korea. The plants were grown under open field conditions at the Breeding Research Institute of Koregon, Gimje, Korea, and harvested at the commercial stage, as confirmed by an expert. Carrot roots were sampled after cleaning the dust and soil. Three carrot roots of each genotype were used. Roots were harvested at the marketable stage. Each root was separated into outer (cortex and phloem) and inner (xylem and pith) parts and stored in a separate bag. The carrot samples were then freeze-dried in a freeze-dryer (FD5508; ilShinBioBase Co. Ltd.; Gyeonggi-do, South Korea) and ground into a fine powder. The powder samples were stored at −20 °C under air-tight conditions until analyzed. Bioactive compounds and antioxidant activities were analyzed within one month of storage.

### 2.3. Analysis of Carotenoids

Three carotenoids (⍺-carotene, 𝛽-carotene, and lutein) were analyzed using high-performance liquid chromatography (HPLC) according to Bhandari et al. [7]. Briefly, 0.2 g of carrot powder was extracted in 5.0 mL of chloroform: MeOH (1:1, *v/v*), diluted with methanol (100%), filtered, and injected into an HPLC system (Agilent Technologies, Santa Clara, CA, USA) equipped with a diode array detector (DAD) set at 470 nm. Carotenoid peaks were separated using a Nova-Pak- C18 4 μm (3.9 × 150 mm) column (Waters, Milford, MA, USA) in an isocratic mobile phase composed of methanol (100%) at a flow rate of 1.5 mL min^−1^. Authentic standards of each carotenoid were used for the identification and quantification of the peaks.

### 2.4. Analysis of Sugar Content and Total Sweetness Index (TSI)

Three major free sugars, namely, glucose, fructose, and sucrose, were analyzed based on the method described by Bhandari et al. [7]. Carrot powder (0.2 g) was extracted in distilled water (5.0 mL) at 80 °C for 20 min, cooled immediately, centrifuged, filtered, and injected into an HPLC system equipped with a refractive index (RI) detector. The peaks were separated using a carbohydrate analysis column (4.6 × 250 mm, 5 µm; ZORBAX, Agilent Technologies) in an isocratic mobile phase composed of acetonitrile/distilled water (75/25, *v/v*) at a flow rate of 1.4 mL min^−1^. Authentic standards for each sugar were used to identify and quantify the peaks. The total sweetness index (TSI) was calculated as described by Magwaza and Opara [30].

### 2.5. Analysis of Ascorbic Acid

Carrot samples (0.5 g) were extracted in 5% metaphosphoric acid solution (5 mL), centrifuged, filtered, and analyzed using an HPLC system, as described by Bhandari and Lee [31]. Separation of the peak was achieved by using an Acquity UPLC HSS T3 (2.1 × 100 mm, 1.8 μm, Waters) column in an isocratic mobile phase composed of aqueous 0.1% (*v/v*) formic acid solution at a flow rate of 0.3 mL min^−1^ and detected using a diode array detector (DAD) at 254 nm. L-ascorbic acid standards at various concentrations (0–100 ppm) were used for the identification and quantification of the peak.

### 2.6. Analysis of Vitamin E and Phytosterols

Sample preparation and analysis of vitamin E, squalene, and phytosterols were performed according to the method described by Bhandari et al. [32], with some modifications. Freeze-dried carrot powder (0.2 g) was mixed with ethanol and ascorbic acid, and extracted in a water bath for 18 min at 80 °C. Then, 300 µL of 44% KOH was added, and the mixture was shaken for 10 min for saponification. Ten milliliters of n-hexane and 10 mL of dH2O were added, vortexed, and centrifuged at 3500 rpm for 10 min, and the upper hexane layer was collected. This process was repeated thrice, and the collected hexane samples were pooled and washed twice with 10 mL dH2O. The samples were then passed through anhydrous sodium sulfate (Na_2_SO_4_) to remove water, concentrated in a rotary evaporator, dissolved in 1 mL of iso-octane, and left overnight at room temperature. The sample was then injected into a gas chromatograph (SHIMADZU GC-2010 Plus) equipped with a flame ionization detector (FID) and capillary column (DB-5; 100 m × 0.25 mm, thickness: 0.25 µm). The injector and detector were set at 290 and 320 °C, respectively, and the injection volume was 1 µL with a split ratio of 1:20 at a constant column flow of 0.40 mL min^−1^ with helium as the carrier gas. The oven temperature was set to 250 °C for 2 min, increased to 281 °C at a rate of 4 °C min^−1^, held constant for 30 min, increased to 315 °C at a rate of 10 °C min^−1^, and held constant for 10 min. Different concentrations of authentic vitamin E standards were used to identify and quantify each vitamin E isomer.

### 2.7. Analysis of Fatty Acid Composition

The fatty acid composition was analyzed according to Bhandari et al. [32], with some modifications. Powdered carrot samples (0.1 g) were extracted with 680 μL of a methylation mixture [methanol (MeOH):benzene:2,2-dimethoxypropane:H_2_SO_4_ = 39:20:5:2] and 400 μL of heptane for 2 h at 80 °C in a water bath, and then cooled to room temperature. The heptane layer was then collected and injected into a GC equipped with a flame ionization detector and a capillary column (SP 2560; 100 m × 0.25 mm, thickness: 0.2 µm). The injector and detector temperatures were set at 250 °C and 260 °C, respectively. The injection volume was 1 μL, with a split ratio of 1:20 at a constant column flow rate of 1.08 mL min^−1^. The oven temperature was initially maintained at 120 °C for 2 min, increased to 240 °C at a rate of 4 °C min^−1^, and held constant for 23 min. A mixture of 19 fatty acid methyl esters was used to identify the fatty acids.

### 2.8. Measurement of Total Phenol, Total Flavonoid, and Total Anthocyanin Content

Carrot samples (0.05 g) were extracted with 80% methanol for 1 h at 50 °C in a water bath at 150 rpm, centrifuged at 10,000 rpm, and filtered. The total phenolic content (TPC) was estimated using the Folin–Ciocalteu colorimetric method, as described by Bhandari et al. [33]. Two hundred microliters of the supernatant and 0.6 mL of distilled water were added to a 2.0-mL Eppendorf tube. After adding 200 µL of Folin’s reagent, the reaction mixture was vortexed and incubated in a water bath at 27 °C for 5 min. Then, 200 µL of 15% Na_2_CO_3_ solution was added to the mixture and incubated for 1 h in the dark at room temperature (25 °C). Then, the solution was centrifuged at 12,000 rpm for 10 min at 4 °C. The absorbance of 200 µL of the reaction mixture was then measured at 760 nm using a microplate reader (Multiskan GO, Thermo Fisher Scientific Inc., Waltham, MA, USA). Gallic acid at various concentrations (0.0–200.0 ppm) was used to generate a standard curve.

Total flavonoid content (TFC) was measured using the colorimetric method described by Bhandari et al. [34]. Two hundred microliters of aliquot (same aliquot obtained for TPC analysis) and 800 μL water were mixed in a 2 mL Eppendorf tube, followed by the addition of 60 μL NaNO_2_ (5%). Then, 60 μL of AlCl_3_6H_2_O (10%) and 400 μL of NaOH (1M) were added simultaneously after 5 min, and the absorbance of the reaction mixture (200 µL) was measured at 510 nm using a microplate reader. Catechin hydrate at different concentrations (10–200 ppm) was used to generate the standard curve.

Total anthocyanin content was measured according to the method described by Lazcano et al. [35], with some modifications. Briefly, 100 mg of powdered sample was mixed in 1 mL buffer (1N HCl), vortexed, and centrifuged, and the upper layer was collected. The residue was re-extracted in the same solution, and the aliquot was mixed. Then, 800 µL of the sample aliquot and 400 µL of n-hexane were mixed, vortexed, and centrifuged. The absorbance of the lower layer of the solution was measured at 535 nm, and the total anthocyanin content was calculated from the extinction coefficient.

### 2.9. Measurement of Antioxidant Activities

The antioxidant activities of carrots were evaluated using two different methods, namely, ferric-reducing antioxidant power (FRAP) and 2,2′-azino-bis (3-ethylbenzothiazoline-6sulfonic acid) [ABTS] assays. The FRAP assay was performed as described by Bhandari et al. [33]. First, 300 mM acetate buffer (pH 3.6), 10 mM TPTZ in 40 mM HCl, and 20 mM FeCl_3_∙6H_2_O were prepared and mixed in a 10:1:1 ratio (*v/v/v*) to prepare the FRAP working solution. The same aliquot obtained for TPC analysis (50 µL) was mixed with 950 µL of the FRAP working solution and heated for 10 min at 37 °C. The absorbance of the reaction mixture (200 µL) was measured at 593 nm using a microplate reader. Trolox at different concentrations (0–1000 µmol) was used to calculate the standard curve. The ABTS assay was performed as described by Bhandari et al. [33]. First, 7 mM ABTS solution and 2.45 mM potassium persulfate were mixed and kept in the dark for 16 h at 25 °C to produce ABTS radical cations (ABTS^●+^). The mixture was diluted with methanol to achieve an absorbance of 0.9 ± 0.02 at 734 nm. The sample aliquot (50 μL; the same aliquot obtained for TPC analysis) was then added to 950 μL of ABTS^●+^ solution and kept in the dark for 2 h. The absorbance of the reaction mixture (200 µL) was measured at 734 nm using a microplate reader. Different concentrations of Trolox (100–1000 μmol L^−1^)) were used as standards to calculate the standard curve.

### 2.10. Statistical Analyses

The mean of three biological replicates was used for statistical analyses using SPSS Statistics 20.0 (ver. 20; IBM, Armonk, NY, USA). Analysis of variance followed by Duncan’s multiple range tests were used to analyze the statistical differences among the means at *p* > 0.05. Correlation analysis was performed using Pearson’s correlation coefficient (r) at *p* < 0.05. Figures were generated using SigmaPlot^®^12 (Systat Software Inc., San Jose, CA, USA).

## 3. Results

### 3.1. Variation in Carotenoid Content

Carotenoid content in different genotypes of carrot varied significantly in the outer and inner tissues and was highly correlated with root color (Table 1). Genotypes with orange roots contained all three carotenoids analyzed in this study: lutein, α-carotene, and β-carotene. In contrast, white carrots exhibited only lutein content, while purple carrots exhibited lutein and β-carotene. White carrots exhibited significantly higher lutein content in the outer tissues than in the inner tissues, while the accumulation of lutein in orange and purple carrots was dependent on the genotype. Alpha- and β-carotene contents were higher in the outer tissues of orange roots than in the inner tissues. Furthermore, β-carotene was also higher in the outer tissues than in the inner tissues in all genotypes of orange and purple carrots. Total carotenoid content was significantly higher in the outer tissues than in the inner tissues of all genotypes, regardless of root color. Among the nine genotypes, genotypes with orange roots (n = 3) had relatively higher total carotenoid content (1.57 to 1.70 mg g^−1^ in outer and 0.54 to 0.72 mg g^−1^ in inner root tissue). Overall, K263794 had the highest total carotenoid content in both inner (0.72 mg g^−1^) and outer (1.70 mg g^−1^) tissues.

### 3.2. Variation in Free Sugar Content and Total Sweetness Index (TSI)

The levels of fructose, glucose, and sucrose were analyzed in both the outer and inner tissues of the genotypes (Table 2). We found relatively higher sucrose content in outer tissues (146.9 to 291.0 mg g^−1^) than in inner ones (135.5 to 277.6 mg g^−1^) in all genotypes. Fructose content was significantly higher or similar between the inner and outer tissues depending on the genotype. In contrast, glucose content was highly dependent on the genotypes showing statistically higher, similar, or lower values in the outer tissues than in the inner ones.

Among the different root colors, orange and white root carrots exhibited relatively higher glucose contents in their respective inner and outer tissues. Fructose and sucrose content in the outer and inner tissues were dependent on the genotype of the plant. The total sugar content exhibited a similar pattern to that of sucrose content in all genotypes. It ranged from 251.7 to 370.6 mg g^−1^ and 295.3 to 433.8 mg g^−1^ in inner and outer tissues, respectively. The highest total sugar content in inner and outer tissues was found in genotypes ‘201171′ and ‘K263794′, both of which had orange roots. Overall, our results showed significant variations in individual and total sugar content with root color, tissue position, and genotype, with sugar content being higher in outer tissues than in inner tissues in all genotypes. The total sweetness index (TSI) also followed the same pattern as the total sugar content in the outer and inner tissues of all the genotypes.

### 3.3. Variation in Ascorbic Acid, Total Anthocyanin, Total Phenol, and Total Flavonoid Content

**Figure 1:**
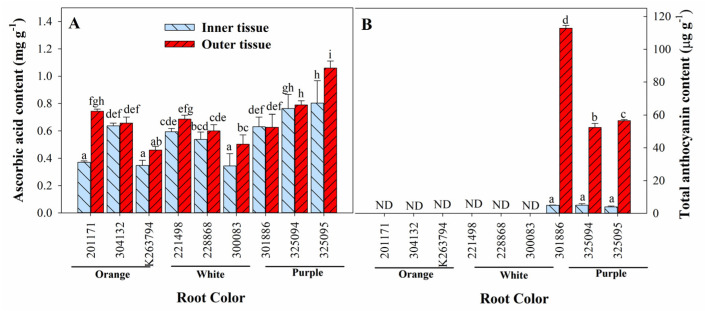
Tissue-dependent variation in ascorbic acid (**A**) and total anthocyanin (**B**) content of carrot roots with different colors. Each bar represents the mean ± standard deviation (SD) of three replicates. Different letters indicate statistically significant differences according to Duncan’s multiple range test at *p* < 0.05. ND: not detected.

Ascorbic acid content also exhibited tissue-, root-, and genotype-dependent variation. Outer tissues exhibited relatively higher ascorbic acid content than inner tissues in all genotypes (Figure 1A). It ranged from 0.35 to 0.80 mg g^−1^ and 0.46 to 1.06 mg g^−1^ in inner and outer tissues, respectively. Generally, genotypes with purple roots had relatively higher ascorbic acid content than those with orange and white roots. The highest ascorbic acid content was found in ‘325095′, a purple carrot in both the inner and outer tissues. Total anthocyanin content was found only in purple carrots (Figure 1B) and was significantly higher in outer tissues than inner tissues. The highest total anthocyanin content was found in ‘301886′ followed by ‘325095′ and ‘325094′.

**Figure 2:**
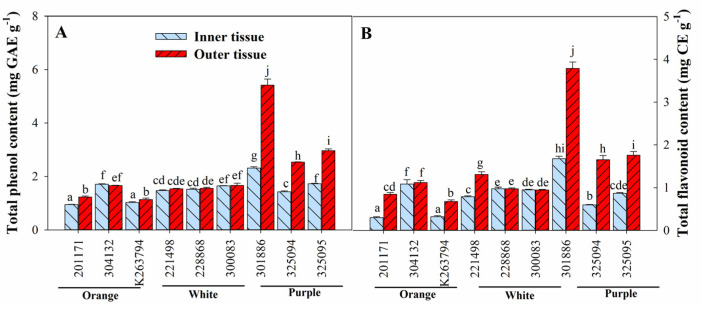
Tissue-dependent variation in total phenol (**A**) and total flavonoid (**B**) content of carrot roots with different colors. Each bar represents the mean ± standard deviation (SD) of three replicates. Different letters indicate statistically significant differences according to Duncan’s multiple range test at *p* < 0.05.

Total phenol content (TPC) and total flavonoid content (TFC) also exhibited similar accumulation patterns to ascorbic acid. Genotypes exhibiting orange and white roots had similar or significantly higher TPC and TFC content in outer tissues than in inner tissues, depending on the genotypes, while genotypes with purple roots had significantly higher TPC and TFC content in outer tissues than in inner ones (Figure 2).

Moreover, carrots with purple roots had relatively higher TPC and TFC than the other root color genotypes. Among the different genotypes, ‘301866′ with purple roots had the highest TPC (inner and outer tissues: 2.31 and 5.42 mg GAE g^−1^, respectively) and TFC (inner and outer tissues: 1.68 and 3.79 mg CE g^−1^, respectively), suggesting the superiority of these genotypes in term of antioxidant content.

### 3.4. Variation in Vitamin E and Phytosterols Content

Among the eight vitamin E isomers analyzed, only four vitamin E isomers, α-tocopherol, γ-tocopherol, α-tocotrienol, and γ-tocotrienol, were detected in carrot roots (Table 3). α-tocopherol was a major vitamin E isomer found in all carrot genotypes in both outer and inner tissues. It ranged from 15.99 to 48.78 µg g^−1^ and 24.22 to 61.17 µg g^−1^ in inner and outer tissues, respectively. The outer tissues exhibited significantly higher α-tocopherol content than the inner tissues of all genotypes. Genotypes with purple roots generally had higher α-tocopherol content than those with other root colors. Alpha-tocotrienol was the second most dominant vitamin E isomer found in all the carrot genotypes except in ‘221498′, a carrot with white roots. The tissue-dependent accumulation of α-tocotrienol was highly dependent on genotype. γ-tocopherol was detected only in white carrot genotypes, and its level was much lower when compared to that of α-tocopherol and tocotrienol. Furthermore, γ-tocopherol content was highly dependent on the genotypes exhibiting statistically higher or lower content in outer tissues than in inner tissues. γ-tocotrienol was the least dominant vitamin E isomer in this study, which was only found in two genotypes of white carrots. It exhibited inconsistent tissue-dependent accumulation. The total vitamin E content was statistically higher in the outer tissues than in the inner tissues of almost all genotypes. It ranged from 19.19 µg g^−1^ in the inner tissue of the white carrot (‘221498′) to 67.51 µg g^−1^ in the outer tissue of the purple carrot (‘301886′). Overall, genotypes with purple roots had relatively higher total vitamin E content than genotypes with other root colors. The ‘301886′ genotype exhibited the highest total vitamin E content in the inner (57.14 µg g^−1^) and outer tissues (67.51 µg g^−1^) among the genotypes used in this study.

Among the three phytosterols analyzed in this study, β-sitosterol was the most dominant phytosterol and accounted for 73.9–81.6% of the total phytosterol content, followed by stigmasterol and campesterol in all genotypes and their respective tissues (Table 4). β-sitosterol content ranged from 373.5 µg g^−1^ in the inner tissue of ‘304132′ to 541.2 µg g^−1^ in the outer tissue of ‘201171′, both exhibiting orange roots. In orange- and purple-colored genotypes, β-sitosterol content was relatively higher in the outer tissues than in the inner tissues, showing significant and non-significant results in orange- and purple-colored carrots, respectively. In contrast, white-colored carrots exhibited inconsistent results depending on the genotype. The highest β-sitosterol content in inner and outer tissues was found in ‘221498′ (498.8 µg g^−1^) and ‘201171′ (541.2 µg g^−1^), respectively. Stigmasterol also exhibited an accumulation pattern similar to that of β-sitosterol. The highest stigmasterol content in inner and outer tissues was found in ‘300083′ (89.9 µg g^−1^) and ‘201171′ (106.4 µg g^−1^), respectively. Campesterol also exhibited the most variation among the genotypes and their tissues in most cases. The highest and lowest campesterol levels were found in the outer tissue of ‘228868′ (86.1 µg g^−1^) and the inner tissue of ‘K263794′ (44.0 µg g^−1^), respectively. Total phytosterol content ranged from 471.0 µg g^−1^ in the inner tissue of ‘304132′ to 707.3 µg g^−1^ in the outer tissue of ‘201171′. Genotypes with orange and purple roots exhibited statistically higher total phytosterol content in the outer tissues than in the inner tissues. In contrast, the accumulation of total phytosterol content was dependent on the genotype in the white carrots. However, genetic variation did not depend on root color as much as it did for carotenoid content.

### 3.5. Variation in Fatty Acid Composition

Among the 19 fatty acids analyzed in this study, only 14 were detected, comprising 8 saturated and 6 unsaturated fatty acids (Table 5). Unsaturated fatty acids accounted for a greater portion of total fatty content (>70%) than saturated fatty acids (<30%). One saturated fatty acid [palmitic (18.6–23.7% of total fatty acid composition)] and two unsaturated fatty acids [linoleic (60.5–70.7%) and linolenic acid (4.8–10.8%)] were the major fatty acids, accounting for >90.0% of total fatty acids. Other fatty acids included lauric, myristic, pentadecanoic, palmitoleic, heptadecanoic, stearic, oleic, arachidic, eicosenoic, behenic, and erucic acids. Among the three major fatty acids, linoleic acid had a relatively higher abundance in the outer tissues (62.3–70.7%) than in the inner tissues (60.5–66.6%), regardless of root color and genotype. In contrast, palmitic and linolenic acid exhibited a reverse trend, with higher values in the inner tissues than in the outer tissues in all genotypes. Other fatty acids exhibited inconsistent accumulation patterns depending on the genotype, root color, and tissue.

### 3.6. Variation in Antioxidant Activities

**Figure 3:**
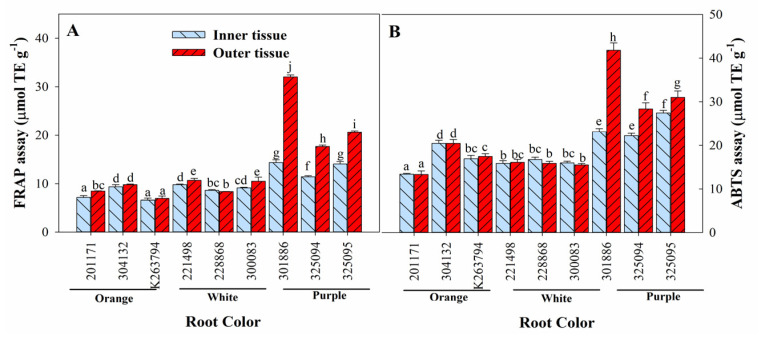
Tissue-dependent variation in antioxidant activity ((**A**) FRAP assay and (**B**) ABTS assay) of carrot roots with different colors. Each bar represents the mean ± standard deviation (SD) of three replicates. Different letters indicate statistically significant differences according to Duncan’s multiple range test at *p* < 0.05. FRAP: ferric reducing antioxidant power; ABTS: 2,2′-azinobis(3-ethylbenzothialozine-6-sulfonic acid).

Antioxidant activities were evaluated using FRAP and ABTS assays (Figure 3). Both assays revealed similar trends between the inner and outer tissues of all the genotypes. Outer tissues exhibited similar or higher antioxidant activity than inner tissues, depending on the genotype. Genotypes with purple roots exhibited significantly higher antioxidant activity in both inner and inner tissues compared to genotypes with other root colors. The ‘301886′ genotype exhibited the highest FRAP (32.02 µmol TE g^−1^) and ABTS values (41.80 µmol TE g^−1^) in its outer tissues, which was followed by ‘325094′ and ‘325095′.

### 3.7. Correlation Analysis between Chemical Composition and Antioxidant Activities, and Principal Component Analysis (PCA)

Correlation analysis was performed for antioxidants and antioxidant activities to understand the contribution of each antioxidant to total antioxidant activity. As antioxidants were significantly affected by genotype, root color, and tissue position in this study, the correlation between antioxidant and antioxidant activity was also affected (Table 6). The overall results revealed a significant relationship between total phenolics, total carotenoids, total vitamin E, ascorbic acid, total flavonoids, total anthocyanin, and total phytosterols in the two antioxidant assays (ABTS and FRAP assays). TPC exhibited the highest positive correlations with both antioxidant assays (FRAP:0.972 **; ABTS:0.899 **), followed by total anthocyanin, TFC, total vitamin E, and vitamin C. In contrast, total carotenoid and total phytosterol levels were negatively correlated with the FRAP and ABTS assays.

**Figure 4:**
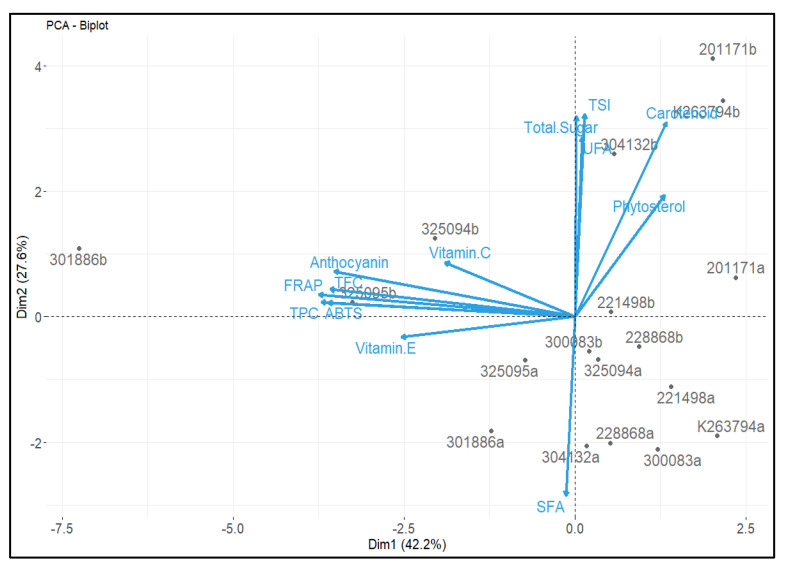
Principal component analysis (PCA) of phytochemicals, fatty acids, and antioxidant activities in the outer and inner tissues of carrot genotypes. The lines starting from the center point of the bi-plot show the positive or negative association of the parameters. The letters a and b after the name of genetic resources are the inner and outer tissues of respective genotypes. TSI: total sweetness index; TPC: total phenol content; TFC: total flavonoid content; SFA: saturated fatty acids; UFA: unsaturated fatty acids; FRAP: ferric reducing antioxidant power; ABTS: 2,2′-azinobis(3-ethylbenzothialozine-6-sulfonic acid).

PCA was conducted to achieve any connection between the analyzed phytochemicals and antioxidant activities in the outer and inner tissues of genetic resources. PCA showed a clear trend of separation among the phytochemicals, genetic resources, and their tissues (inner and outer). We observed the two highest principal components, showing approximately 69.8% of total variations (Figure 4). The first principal component (Dim 1) and second principal component (Dim 2) represent 42.2 and 27.6% of the total variation, respectively. Total carotenoid, total phytosterol, total free sugar, UFA, and TSI were distributed in a positive direction of PC1, while total anthocyanin, TPC, TFC, vitamin C, and antioxidant activities (FRAP and ABTS assay) were distributed in a positive direction of PC2, showing less variation (27.6 *). Total vitamin E and SFA were in a negative distribution of PC2. The results also showed that the outer tissue of ‘301886′ with purple roots had the highest total anthocyanin, total vitamin E, TPC, TFC, and antioxidant activities. We found the highest TSI, total phytosterol, total carotenoid, and total sugar content in the genotype (K263794) with orange roots.

## 4. Discussion

In this study, we evaluated carotenoids, vitamins (C and E), free sugars, phytosterols (β-sitosterol, stigmasterol, and campesterol), fatty acid composition, TPC, total anthocyanin content, TFC, and antioxidant activities (FRAP and ABTS assays) in the inner and outer tissues of nine carrot genotypes with different root colors (orange, white, and purple). The results revealed differential accumulation of the studied compounds depending on the genotype, tissue, and root color. We found considerable amounts of carotenoids, ascorbic acid, TPC, TFC, total anthocyanin, vitamin E, free sugars, and fatty acids, most of which were within the range reported in previous studies [5,7,8,28]. The contribution of each antioxidant to antioxidant activity was also evaluated using correlation analysis, while PCA was performed to identify the suitable genotypes of interest and their root tissues based on the phytochemical contents and antioxidant activities. Genotypes with orange roots had all three carotenoids; purple carrots had only two carotenoids (lutein and β-carotene), and white carrots possessed only lutein. A similar root color-dependent variation in carotenoids was also reported in previous studies [7,8], which might be due to the difference in the expression levels of carotenoid biosynthetic genes [36]. Genotypes with orange roots exhibited statistically higher carotenoid content than other root colors due to the presence of homozygous recessive genes (yyy2y2) located on chromosomes 3 and 7, which are responsible for the higher carotenoid accumulation [37]. Similarly to the findings of Arscott and Tanumihardjo [38], lutein was most dominant in white and purple carrots. Moreover, there was also a clear difference in individual carotenoid content between the outer and inner tissues of respective genotypes, with outer tissues exhibiting greater carotenoid content than inner tissues, consistent with the findings of Koch and Goldman [39]. Such tissue-specific variation in carotenoid accumulation was probably based on the translocation factor of the outer and inner portions, as the outer portion mainly comprises the phloem acting as a sink, while the inner portion composed of xylem is responsible for the movement of minerals and water from the root to the different parts of the plant.

Free sugars also exhibited clear genetic- and tissue-dependent variation. The compositional ratio and content of free sugars and inorganic acids are responsible for increasing the palatability of vegetables and fruits. Fructose is the sweetest free sugar, whereas sucrose is a major transport and storage facilitator of sugar in the underground organs of plants, including carrots [23,30]. Consistent with previous reports [7,40,41], sucrose was the most dominant free sugar, regardless of genotype, root color, and tissue, although some inconsistencies have been observed in previous reports [42]. The total sugar content was higher in the outer tissues than in the inner tissues of all genotypes. Furthermore, TSI was also relatively higher in the outer tissues than in the inner tissues of all the genotypes, suggesting the higher palatability and sweetness of outer tissues than inner tissues.

Ascorbic acid, total anthocyanin content, TPC, and TFC were also affected by genotype, root color, and tissue. Purple-colored genotypes exhibited relatively higher abundances of these antioxidants in both inner and outer tissues. Anthocyanin, which generally accumulates in purple carrots, was found only in purple-colored genotypes, although a previous report also revealed traces of anthocyanin in orange and white carrots [28]. The presence of anthocyanin in purple-colored carrot genetic resources was due to the regulation of genes in the P_1_ and P_3_ regions of the chromosomes [43]. Among the nine genotypes; ‘301886′ with purple roots had significantly higher TPC, TFC, and total anthocyanin content than other genotypes, indicating its higher antioxidant potential, which was also observed through correlation analysis.

Vitamin E is a group of lipophilic antioxidants (α-, β-, γ-, and 𝛿- tocopherols and tocotrienols) that are naturally synthesized in the plastids of plants [44]. Similarly to the study by Luby et al. [18], we found only four vitamin E isomers in this study, namely, α- and γ- tocopherols and α- and γ-tocotrienols, and α-tocopherol was the most dominant vitamin E isomer followed by α-tocotrienol in both the outer and inner tissues of all the genotypes. However, our results were inconsistent with those of Ombodi et al. [45], who found three tocopherols and one tocotrienol in their study. These discrepancies might be due to the differences in plant genotypes. γ- tocopherol and tocotrienol were only found in the genotypes of white carrots. Among the three root colors, orange carrot genotypes exhibited significantly higher total vitamin E content in outer tissues than in inner tissues, while white- and purple-colored genotypes exhibited inconsistent results depending upon the genotype, which was consistent with the observations of Koch and Goldman [39]. Like the carotenoid content, vitamin E isomers also exhibited color-dependent accumulation in outer and inner tissues, which was probably due to the common precursor [geranylgeranyl pyrophosphate (GGPP)] they share during biosynthesis and their antioxidant nature [46]. Among the nine genotypes, ‘301886′ exhibited the highest total vitamin E content in both inner and outer tissues suggesting the high health potential of this genotype as vitamin E contributes significantly to total antioxidant activity.

Phytosterol studies showed the presence of three phytosterols, namely, campesterol, β-sitosterol, and stigmasterol, with β-sitosterol being the major phytosterol, followed by stigmasterol and campesterol. This was consistent with previous reports [19]. Almost all the genotypes exhibited statistically similar or higher total phytosterol content in outer tissues than in inner tissues, suggesting the high health potential of outer tissues as phytosterols help to ameliorate plasma low-density lipoprotein cholesterol levels, non-alcoholic fatty liver disease, and cardiovascular diseases [47,48,49] and also exhibit antioxidant, anti-inflammatory, and antipyretic effects [50,51].

Compositional analysis of the fatty acids revealed the presence of 14 fatty acids. Unlike in carrot seeds, the major fatty acids were palmitic, linoleic, and linolenic acids, comprising >90% of the total fatty acid content [52]. Linoleic and linolenic acids were the major polyunsaturated fatty acids found in all the genotypes and their respective tissues. The genotype- and tissue-dependent variation in fatty acid composition was not as prominent as that of vitamin E and phytosterol content. Although the roots of carrots are not a major source of lipids in food, the high content of polyunsaturated fatty acids in carrot roots may have health potential because these fatty acids contribute to the reduction in cardiovascular risk and low-density lipoprotein (LDL) [53]. To the best of our knowledge, this is the first report to provide information on phytosterol and fatty acid composition in different tissues of carrot roots with different colors.

Two assays, FRAP and ABTS, were used to measure the antioxidant activities, as one method may be insufficient to accurately predict the overall antioxidant capacity. Both assays revealed similar trends in major antioxidant compounds (TPC, TFC, and total anthocyanin content) and ascorbic acid and vitamin E, which was due to the significant contribution of each antioxidant, as observed through correlation analysis. Purple-colored carrots had relatively higher amounts of these antioxidants and exhibited higher antioxidant activity as measured by FRAP and ABTS assays, which is consistent with a previous report [28].

To understand the overall contribution of each antioxidant to antioxidant activity, a correlation analysis was performed. A strong positive correlation was found between antioxidant compounds and antioxidant activity, indicating that the accumulation of these antioxidants was the major cause of the increase in antioxidant activity. The results showed the highest positive correlation of TPC with the FRAP and ABTS assays, followed by total anthocyanin, TFC, and vitamin E, indicating a significant contribution of these compounds to the total antioxidant activity. This has also been observed in a wide range of plants [31,32,33,54]. Non-significant and negative correlation between AA and total phytosterol content, and the least significant correlation between AA and total vitamin E, were also observed, which was probably due to the polarity of the extraction solvent.

Overall, carotenoids, free sugars, and phytosterols were the most abundant in the carrot genotypes with orange roots. The total vitamin E, vitamin C, TPC, TFC, total anthocyanin, and antioxidant activities (FRAP and ABTS assays) were relatively higher in purple carrot genotypes than in orange and white carrots. PCA results clearly discriminated the phytochemicals and antioxidant activities into two groups. Total carotenoid, total phytosterol, total sugar, and TSI were highest in ‘K263794′, while vitamin E, total vitamin E, total anthocyanin, TPC, TFC, and antioxidant activities (FRAP and ABTS assay) were the most dominant in the outer tissues of ‘301886′. In conclusion, this is the first study to provide useful information on a range of bioactive compounds in carrots with different root colors and tissues and their contribution to total antioxidant activity. The overall findings might be useful for the processing and extraction of selected bioactive compounds from the outer tissues of given genotypes for food processing and pharmaceutical industries, respectively.

## Figures and Tables

**Table 1:** Tissue-dependent variation in carotenoid content (mg g^−1^) of carrot genotypes with different root colors.

Root Color	IT Number	Tissue	Lutein	α-Carotene	β-Carotene	Total Carotenoid
Orange	201171	Inner	0.24 ± 0.00 fg	0.16 ± 0.00 b	0.30 ± 0.01 e	0.69 ± 0.01 i
		Outer	0.26 ± 0.01 h	0.48 ± 0.03 e	0.91 ± 0.01 g	1.64 ± 0.02 l
	304132	Inner	0.21 ± 0.01 e	0.08 ± 0.00 a	0.25 ± 0.01 d	0.54 ± 0.02 h
		Outer	0.30 ± 0.01 i	0.33 ± 0.01 d	0.94 ± 0.02 h	1.57 ± 0.03 k
	K263794	Inner	0.14 ± 0.01 b	0.23 ± 0.01 c	0.36 ± 0.02 f	0.72 ± 0.04 j
		Outer	0.12 ± 0.00 a	0.65 ± 0.01 f	0.94 ± 0.02 h	1.70 ± 0.04 m
White	221498	Inner	0.20 ± 0.01 d	ND	ND	0.20 ± 0.01 b
		Outer	0.25 ± 0.01 gh	ND	ND	0.25 ± 0.01 c
	228868	Inner	0.22 ± 0.01 e	ND	ND	0.22 ± 0.01 b
		Outer	0.31 ± 0.01 i	ND	ND	0.31 ± 0.01 de
	300083	Inner	0.16 ± 0.00 c	ND	ND	0.16 ± 0.00 a
		Outer	0.26 ± 0.01 h	ND	ND	0.26 ± 0.01 c
Purple	301886	Inner	0.23 ± 0.01 f	ND	0.03 ± 0.00 a	0.26 ± 0.01 c
		Outer	0.31 ± 0.00 i	ND	0.04 ± 0.00 a	0.35 ± 0.00 f
	325094	Inner	0.26 ± 0.02 h	ND	0.07 ± 0.01 b	0.33 ± 0.03 ef
		Outer	0.26 ± 0.02 h	ND	0.24 ± 0.02 d	0.50 ± 0.02 g
	325095	Inner	0.17 ± 0.01 c	ND	0.04 ± 0.00 a	0.21 ± 0.00 b
		Outer	0.17 ± 0.02 c	ND	0.13 ± 0.00 c	0.29 ± 0.02 d

Values represent the mean ± standard deviation (SD) of three biological replicates. ND: not detected. Different letters in the same column indicate significant differences based on Duncan’s multiple range test at *p* < 0.05.

**Table 2:** Tissue-dependent variation in free sugar content (mg g^−1^) and total sweetness index (TSI) of carrot genotypes with different root colors.

Root Color	IT Number	Tissue	Fructose	Glucose	Sucrose	Total Sugar	TSI
Orange	201171	Inner	86.7 ± 3.1 g	110.3 ± 8.8 k	173.7 ± 10.4 de	370.6 ± 15.4 gh	387.5 ± 14.3 g
		Outer	93.7 ± 4.6 h	97.3 ± 2.6 j	186.8 ± 3.8 ef	377.9 ± 2.5 h	401.4 ± 4.0 g
	304132	Inner	79.5 ± 4.4 f	64.6 ± 1.7 gh	145.2 ± 9.9 ab	289.2 ± 10.9 bc	313.4 ± 12.3 cd
		Outer	85.7 ± 5.1 g	143.0 ± 6.9 l	155.3 ± 5.0 bc	383.9 ± 9.8 h	392.5 ± 10.6 g
	K263794	Inner	67.1 ± 2.9 de	106.7 ± 10.6 k	166.2 ± 10.7 cd	340.0 ± 18.6 ef	347.9 ± 14.7 ef
		Outer	68.8 ± 2.2 e	83.3 ± 1.6 i	281.7 ± 5.6 h	433.8 ± 6.9 i	448.2 ± 6.7 g
White	221498	Inner	62.1 ± 5.4 d	60.7 ± 2.4 fg	150.3 ± 5.0 abc	273.0 ± 11.0 b	289.5 ± 13.0 b
		Outer	67.2 ± 1.9 de	71.5 ± 0.8 h	192.1 ± 7.7 ef	330.8 ± 5.3 ef	347.2 ± 4.6 ef
	228868	Inner	70.4 ± 4.8 e	93.1 ± 7.1 j	135.5 ± 9.8 a	299.0 ± 19.4 a	311.9 ± 19.5 cd
		Outer	85.3 ± 5.0 g	99.4 ± 3.5 j	146.9 ± 7.7 ab	331.7 ± 14.0 cd	350.4 ± 15.1 ef
	300083	Inner	36.9 ± 0.7 b	36.2 ± 1.4 bcd	178.6 ± 4.2 de	251.7 ± 6.2 de	261.4 ± 6.2 a
		Outer	45.0 ± 1.6 c	49.7 ± 0.6 e	200.6 ± 5.1 f	295.3 ± 5.7 fg	305.9 ± 6.2 bc
Purple	301886	Inner	50.8 ± 1.2 c	33.7 ± 1.1 abc	231.8 ± 3.3 g	316.3 ± 1.5 ef	333.6 ± 1.3 de
		Outer	48.8 ± 2.9 c	54.3 ± 2.5 ef	245.3 ± 5.1 g	348.5 ± 4.5 gh	359.9 ± 4.2 f
	325094	Inner	48.3 ± 0.6 c	40.7 ± 2.2 cd	246.5 ± 6.2 g	335.5 ± 7.7 ef	349.9 ± 7.0 ef
		Outer	51.0 ± 4.6 c	42.1 ± 2.5 d	276.7 ± 30.9 h	369.9 ± 29.7 gh	385.3 ± 29.7 g
	325095	Inner	26.1 ± 2.5 a	26.6 ± 1.7 a	277.6 ± 11.8 h	330.3 ± 14.9 ef	336.9 ± 15.9 ef
		Outer	27.2 ± 0.4 a	29.3 ± 2.4 ab	291.0 ± 6.9 h	347.5 ± 9.3 f	354.1 ± 8.9 ef

Values represent the mean ± SD of three biological replicates. Different letters in the same column indicate significant differences based on Duncan’s multiple range test at *p* < 0.05.

**Table 3:** Tissue-dependent variation in vitamin E content of carrot genotypes with different root colors.

Root Color	IT Number	Tissue	Vitamin E (µg g^−1^ DW)				
			γ-T	α-T	γ-T3	α-T3	Total
Orange	201171	Inner	ND	15.99 ± 0.36 a	ND	6.35 ± 0.05 i	22.34 ± 0.40 b
		Outer	ND	24.22 ± 1.84 b	ND	4.66 ± 0.05 f	28.88 ± 1.89 c
	304132	Inner	ND	37.16 ± 1.90 ef	ND	4.26 ± 0.05 e	41.41 ± 1.95 f
		Outer	ND	41.06 ± 0.83 gh	ND	6.25 ± 0.10 i	47.31 ± 0.93 g
	K263794	Inner	ND	16.63 ± 0.96 a	ND	6.53 ± 0.12 j	23.16 ± 0.84 b
		Outer	ND	28.74 ± 1.24 cd	ND	6.82 ± 0.02 k	35.55 ± 1.23 d
White	221498	Inner	1.31 ± 0.03 a	17.89 ± 1.11 a	ND	ND	19.19 ± 1.08 a
		Outer	2.53 ± 0.02 c	26.65 ± 1.02 c	ND	ND	29.18 ± 1.04 c
	228868	Inner	5.15 ± 0.05 d	37.26 ± 0.70 ef	4.90 ± 0.11 d	5.18 ± 0.04 g	52.49 ± 0.82 h
		Outer	1.21 ± 0.03 a	39.28 ± 1.41 fg	2.99 ± 0.22 b	2.95 ± 0.06 c	46.44 ± 1.17 g
	300083	Inner	1.93 ± 0.06 b	27.66 ± 2.20 c	3.33 ± 0.08 c	2.92 ± 0.07 c	35.85 ± 2.11 d
		Outer	9.71 ± 0.34 e	49.83 ± 0.29 j	2.41 ± 0.04 a	4.00 ± 0.05 d	65.95 ± 0.54 j
Purple	301886	Inner	ND	48.78 ± 2.20 j	ND	8.36 ± 0.22 l	57.14 ± 2.42 i
		Outer	ND	61.17 ± 0.93 k	ND	6.34 ± 0.13 i	67.51 ± 1.06 j
	325094	Inner	ND	30.07 ± 0.62 d	ND	5.94 ± 0.16 h	36.01 ± 0.78 d
		Outer	ND	36.46 ± 0.82 e	ND	2.04 ± 0.09 b	38.50 ± 0.87e
	325095	Inner	ND	42.21 ± 1.03 h	ND	4.59 ± 0.04 f	46.79 ± 1.07 g
		Outer	ND	45.44 ± 0.95 i	ND	1.81 ± 0.02 a	47.25 ± 0.96 g

Values represent the mean ± SD of three biological replicates. Different letters in the same column indicate significant differences based on Duncan’s multiple range test at *p* < 0.05. ND: not detected; T: tocopherol; T3: tocotrienol.

**Table 4:** Tissue-dependent variation in phytosterols content of carrot genotypes with different root colors.

Root Color	IT Number	Tissue	Phytosterols (µg g^−1^ DW)			
			β-Sitosterol	Stigmasterol	Campesterol	Total Phytosterol
Orange	201171	Inner	432.7 ± 7.3 d	52.8 ± 2.4 a	44.7 ± 1.4 a	530.2 ± 3.5 bc
		Outer	541.2 ± 5.8 g	106.4 ± 5.1 h	59.7 ± 2.7 fg	707.3 ± 8.3 g
	304132	Inner	373.5 ± 7.1 a	53.3 ± 2.1 a	44.3 ± 1.4 a	471.0 ± 3.7 a
		Outer	410.5 ± 8.2 bc	78.7 ± 3.0 ef	60.6 ± 1.9 g	549.8 ± 13.1 d
	K263794	Inner	410.4 ± 5.0 bc	59.6 ± 0.7 b	44.0 ± 0.2 a	513.9 ± 4.2 b
		Outer	538.4 ± 7.3 g	90.0 ± 1.3 g	55.3 ± 3.0 de	683.7 ± 5.5 f
White	221498	Inner	498.8 ± 8.5 f	66.8 ± 1.6 cd	58.7 ± 1.0 efg	624.2 ± 11.0 e
		Outer	413.3 ± 7.5 bc	55.2 ± 2.8 ab	45.8 ± 2.1 a	514.3 ± 12.4 b
	228868	Inner	411.6 ± 6.0 bc	58.8 ± 3.4 b	79.6 ± 0.6 j	550.0 ± 8.8 d
		Outer	504.8 ± 17.7 f	78.0 ± 2.4 ef	86.1 ± 1.9 k	668.9 ± 17.0 f
	300083	Inner	466.8 ± 6.6 e	89.9 ± 4.6 g	69.3 ± 1.0 i	626.1 ± 10.3 e
		Outer	383.9 ± 8.5 a	70.5 ± 1.3 cd	65.1 ± 2.7 h	519.5 ± 12.6 b
Purple	301886	Inner	401.4 ± 5.9 b	58.4 ± 2.9 b	55.8 ± 3.5 de	515.5 ± 12.4 b
		Outer	410.4 ± 7.2 bc	75.8 ± 2.5 e	57.2 ± 1.7 ef	543.4 ± 6.4 cd
	325094	Inner	419.4 ± 9.3 cd	78.1 ± 2.6 ef	53.6 ± 2.0 cd	551.2 ± 9.9 d
		Outer	422.6 ± 7.0 cd	65.9 ± 3.5 c	49.7 ± 1.2 b	538.3 ± 11.6 cd
	325095	Inner	398.7 ± 4.1 b	71.1 ± 1.9 d	50.6 ± 0.6 bc	520.4 ± 5.3 b
		Outer	409.9 ± 9.8 bc	81.6 ± 2.3 f	49.1 ± 0.7 b	540.6 ± 12.9 cd

Values represent the mean ± SD of three biological replicates. Different letters in the same column indicate significant differences based on Duncan’s multiple range test at *p* < 0.05.

**Table 5:** Tissue-dependent variation in major fatty acid composition (%) of carrot genotypes with different root colors.

Root Color	IT Number	Tissue	Fatty Acid Composition (%)														SFA	UFA
			C12:0	C14:0	C15:0	C16:0	C17:0	C18:0	C20:0	C22:0	C16:1	C18:1	C18:2	C18:3	C20:1	C22:1		
Orange	201171	Inner	0.06 ± 0.00	0.21 ± 0.01	0.34 ± 0.02	21.35 ± 0.23	0.32 ± 0.01	0.70 ± 0.02	0.37 ± 0.02	0.71 ± 0.02	0.88 ± 0.04	1.17 ± 0.02	66.60 ± 0.40	6.88 ± 0.06	0.22 ± 0.00	0.19 ± 0.02	25.03 ± 0.29	75.00 ± 0.30
		Outer	0.06 ± 0.01	0.19 ± 0.01	0.32 ± 0.01	18.57 ± 0.44	0.28 ± 0.01	0.66 ± 0.02	0.47 ± 0.02	0.60 ± 0.02	1.07 ± 0.03	1.89 ± 0.02	70.70 ± 0.49	4.80 ± 0.03	0.18 ± 0.01	0.20 ± 0.01	23.07 ± 0.45	76.93 ± 0.45
	304132	Inner	0.10 ± 0.02	0.27 ± 0.02	0.40 ± 0.02	22.66 ± 0.13	0.55 ± 0.02	0.87 ± 0.01	0.48 ± 0.01	0.69 ± 0.02	0.44 ± 0.01	0.54 ± 0.01	63.49 ± 0.10	8.93 ± 0.02	0.45 ± 0.01	0.13 ± 0.02	25.80 ± 0.10	74.20 ± 0.10
		Outer	0.06 ± 0.01	0.16 ± 0.01	0.33 ± 0.01	19.39 ± 0.13	0.33 ± 0.01	0.84 ± 0.01	0.69 ± 0.04	0.82 ± 0.02	1.06 ± 0.04	1.32 ± 0.04	69.57 ± 0.02	5.16 ± 0.04	0.17 ± 0.01	0.09 ± 0.01	23.50 ± 0.10	76.50 ± 0.10
	K263794	Inner	0.08 ± 0.01	0.20 ± 0.01	0.59 ± 0.01	23.60 ± 0.06	0.67 ± 0.01	0.87 ± 0.03	0.50 ± 0.06	0.99 ± 0.01	0.91 ± 0.01	1.11 ± 0.02	62.83 ± 0.10	6.91 ± 0.01	0.55 ± 0.01	0.20 ± 0.01	28.03 ± 0.06	71.97 ± 0.06
		Outer	0.09 ± 0.01	0.19 ± 0.01	0.48 ± 0.01	20.06 ± 0.19	0.46 ± 0.01	1.11 ± 0.06	0.73 ± 0.01	0.85 ± 0.04	1.45 ± 0.01	1.39 ± 0.02	67.35 ± 0.10	5.46 ± 0.09	0.20 ± 0.01	0.18 ± 0.02	25.23 ± 0.25	74.77 ± 0.25
White	221498	Inner	0.07 ± 0.00	0.17 ± 0.01	0.40 ± 0.02	21.78 ± 0.18	0.37 ± 0.01	0.34 ± 0.03	0.36 ± 0.03	0.54 ± 0.02	0.60 ± 0.06	1.97 ± 0.02	65.60 ± 0.35	7.51 ± 0.08	0.22 ± 0.02	0.07 ± 0.01	25.67 ± 0.25	74.33 ± 0.25
		Outer	0.07 ± 0.00	0.17 ± 0.01	0.32 ± 0.01	20.69 ± 0.13	0.31 ± 0.03	0.51 ± 0.03	0.61 ± 0.02	0.68 ± 0.01	1.04 ± 0.06	3.26 ± 0.13	66.82 ± 0.37	5.19 ± 0.02	0.25 ± 0.02	0.06 ± 0.01	26.37 ± 0.35	73.63 ± 0.35
	228868	Inner	0.07 ± 0.01	0.17 ± 0.01	0.43 ± 0.02	23.50 ± 1.29	0.38 ± 0.02	0.51 ± 0.04	0.39 ± 0.02	0.46 ± 0.02	0.93 ± 0.02	1.09 ± 0.03	63.26 ± 1.20	8.55 ± 0.10	0.18 ± 0.01	0.08 ± 0.01	27.10 ± 1.30	72.90 ± 1.30
		Outer	0.10 ± 0.02	0.24 ± 0.02	0.40 ± 0.02	22.07 ± 0.92	0.39 ± 0.03	1.07 ± 0.02	0.58 ± 0.02	0.65 ± 0.00	1.19 ± 0.03	1.17 ± 0.02	63.16 ± 0.92	8.68 ± 0.02	0.16 ± 0.02	0.15 ± 0.01	26.63 ± 0.95	73.37 ± 0.95
	300083	Inner	0.07 ± 0.00	0.21 ± 0.01	0.40 ± 0.03	21.81 ± 1.02	0.42 ± 0.03	0.64 ± 0.01	0.41 ± 0.04	0.61 ± 0.03	0.79 ± 0.07	1.28 ± 0.03	64.44 ± 0.95	8.58 ± 0.04	0.16 ± 0.01	0.18 ± 0.02	25.63 ± 0.95	74.40 ± 1.00
		Outer	0.06 ± 0.01	0.25 ± 0.02	0.32 ± 0.03	19.61 ± 0.88	0.30 ± 0.02	0.91 ± 0.02	0.53 ± 0.02	0.62 ± 0.01	1.12 ± 0.02	2.54 ± 0.04	67.61 ± 0.97	5.86 ± 0.11	0.20 ± 0.02	0.06 ± 0.01	25.13 ± 0.85	74.87 ± 0.86
Purple	301886	Inner	0.06 ± 0.00	0.21 ± 0.03	0.43 ± 0.01	23.65 ± 0.70	0.46 ± 0.03	0.64 ± 0.02	0.41 ± 0.03	0.67 ± 0.02	0.48 ± 0.02	0.89 ± 0.05	60.45 ± 0.94	10.76 ± 0.21	0.75 ± 0.03	0.15 ± 0.01	26.80 ± 0.80	73.20 ± 0.80
		Outer	0.07 ± 0.01	0.19 ± 0.01	0.33 ± 0.01	20.98 ± 0.26	0.33 ± 0.03	0.64 ± 0.02	0.56 ± 0.11	0.84 ± 0.03	0.93 ± 0.02	1.88 ± 0.08	62.25 ± 0.24	10.74 ± 0.25	0.18 ± 0.01	0.09 ± 0.01	25.33 ± 0.15	74.67 ± 0.14
	325094	Inner	0.09 ± 0.00	0.15 ± 0.01	0.36 ± 0.03	22.25 ± 0.79	0.50 ± 0.01	0.79 ± 0.02	0.43 ± 0.02	0.85 ± 0.02	0.65 ± 0.02	1.08 ± 0.03	64.95 ± 0.95	7.39 ± 0.17	0.33 ± 0.02	0.17 ± 0.01	25.87 ± 0.75	74.13 ± 0.74
		Outer	0.08 ± 0.00	0.13 ± 0.01	0.29 ± 0.01	19.68 ± 0.68	0.36 ± 0.03	1.47 ± 0.06	0.84 ± 0.05	1.05 ± 0.05	1.19 ± 0.04	1.94 ± 0.02	67.81 ± 0.81	4.83 ± 0.02	0.21 ± 0.01	0.13 ± 0.02	25.13 ± 0.64	74.90 ± 0.70
	325095	Inner	0.07 ± 0.01	0.18 ± 0.01	0.42 ± 0.01	21.92 ± 0.48	0.42 ± 0.02	0.70 ± 0.03	0.46 ± 0.01	0.61 ± 0.07	0.88 ± 0.06	1.00 ± 0.05	64.68 ± 0.71	8.28 ± 0.09	0.21 ± 0.01	0.17 ± 0.01	25.57 ± 0.55	74.43 ± 0.56
		Outer	0.06 ± 0.00	0.17 ± 0.01	0.29 ± 0.02	21.17 ± 0.93	0.32 ± 0.01	1.25 ± 0.03	0.81 ± 0.02	0.88 ± 0.02	1.30 ± 0.02	1.77 ± 0.01	66.22 ± 0.99	5.47 ± 0.10	0.17 ± 0.01	0.12 ± 0.02	26.33 ± 0.85	73.67 ± 0.83
F-Value			9.19	25.85	65.26	16.47	79.43	306.8	46.71	105.02	182.89	678.99	43.56	1033.08	476.25	53.15	10.06	9.86
Significance			***	***	***	***	***	***	***	***	***	***	***	***	***	***	***	***

Values represent the mean ± standard deviation (SD) of three replicates. *** indicates significance at *p* < 0.001. C12:0 (lauric acid); C14:0 (myristic acid); C15:0 (pentadecanoic acid); C16:0 (palmitic acid); C17:0 (heptadecanoic acid); C18:0 (stearic acid); C20:0 (arachidic acid); C22:0 (behenic acid); C16:1 (palmitoleic acid); C18:1 (oleic acid); C18:2 (linoleic acid); C18:3 (linolenic acid); C20:1 (eicosenoic acid); C22:1 (erucic acid). SFA: saturated fatty acid; UFA: unsaturated fatty acid.

**Table 6:** Correlation between antioxidants and antioxidant activities in carrots.

Antioxidants	FRAP	ABTS
Ascorbic acid	0.472 **	0.504 **
Total anthocyanin	0.952 **	0.893 **
Total phenol	0.972 **	0.899 **
Total flavonoid	0.930 **	0.833 **
Total carotenoid	−0.300 *	−0.234
Total phytosterol	−0.261	−0.337 *
Total vitamin E	0.565 **	0.541 **

*,** Significant at *p* < 0.05 and 0.01, respectively using Pearson’s correlation analysis. FRAP: ferric reducing antioxidant power; ABTS:2,2′-azinobis(3-ethylbenzothialozine-6-sulfonic acid).

## Data Availability

The data presented in this study are available on request from the corresponding author.
